# Modulation of SIV and HIV DNA Vaccine Immunity by Fas-FasL Signaling

**DOI:** 10.3390/v7031429

**Published:** 2015-03-23

**Authors:** Jiabin Yan, Juan Carlos Zapata, Charles David Pauza, Maria S. Salvato

**Affiliations:** Institute of Human Virology, University of Maryland School of Medicine, Baltimore, MD, 21201, USA; E-Mails: bjyan2004@gmail.com (J.Y.); jczapata@ihv.umaryland.edu (J.C.Z.); cdpauza@ihv.umaryland.edu (C.D.P.)

**Keywords:** DNA vaccine, cell-mediated immunity, SIVgag, HIVenv, Fas-FasL signaling, LCMV

## Abstract

Signaling through the Fas/Apo-1/CD95 death receptor is known to affect virus-specific cell-mediated immune (CMI) responses. We tested whether modulating the Fas-apoptotic pathway can enhance immune responses to DNA vaccination or lymphocytic choriomeningitis virus (LCMV) infection. Mice were electroporated with plasmids expressing a variety of pro- or anti-apoptotic molecules related to Fas signaling and then either LCMV-infected or injected with plasmid DNA expressing SIV or HIV antigens. Whereas Fas or FasL knockout mice had improved CMI, down-regulation of Fas or FasL by shRNA or antibody failed to improve CMI and was accompanied by increases in regulatory T cells (Treg). Two “adjuvant” plasmids were discovered that significantly enhanced plasmid immunizations. The adjuvant effects of Fas-associated death domain (FADD) and of cellular FLICE-inhibitory protein (cFLIP) were consistently accompanied by increased effector memory T lymphocytes and increased T cell proliferation. This adjuvant effect was also observed when comparing murine infections with LCMV-Armstrong and its persisting variant LCMV-Clone 13. LCMV-Armstrong was cleared in 100% of mice nine days after infection, while LCMV-Clone 13 persisted in all mice. However, half of the mice pre-electroporated with FADD or cFLIP plasmids were able to clear LCMV-Clone 13 by day nine, and, in the case of cFLIP, increased viral clearance was accompanied by higher CMI. Our studies imply that molecules in the Fas pathway are likely to affect a number of events in addition to the apoptosis of cells involved in immunity.

## 1. Introduction

New developments in DNA injection technology have improved efficacy for vaccination, adjuvant delivery and gene therapy. DNA vaccines are increasingly attractive for preventing or even treating infectious diseases and cancers, despite a history of low immunogenicity during human clinical trials [1,2,3]. Since DNA vaccines can trigger both innate and adaptive immunity [4], they are suitable when the correlates of protection are uncertain. The recent licensure of four veterinary DNA vaccines [2], and new advances in DNA vaccine technology, especially electroporation [5], have prompted many new clinical trials for infectious diseases, e.g., malaria, HIV, HPV, and cancer, e.g., melanoma, prostate cancer and breast cancer [2]. Best *et al.* [6] showed that electroporation-mediated intramuscular delivery of HPV DNA vaccine induces better immunity than epidermal gene gun-mediated particle delivery. Hirao *et al.* [7] compared the Merck adenovirus serotype 5 (Ad5) SIV vaccine with an optimized electroporation-delivered SIV DNA vaccine in macaques, and concluded that the DNA vaccine induced much better cellular immune responses than did the Ad5 vector. Furthermore, the Ad5 immunization failed to boost following the first immunization while DNA responses were continually boosted with all four immunizations demonstrating a major advantage of the electroporated DNA. Besides the improved delivery, co-delivery of a variety of molecular adjuvants [8,9,10] also greatly improves DNA vaccine potency.

It is well known that the Fas-FasL apoptotic-signaling pathway is responsible for the destruction of antigen-presenting and uninfected bystander cells [11,12]. Here we tested whether modulating the Fas-FasL pathway can enhance HIV DNA vaccine potency. Studies from Norman Letvin’s group had shown that plasmid DNA vaccine-elicited cellular immune responses to HIV Gag were limited by Fas-mediated apoptosis [13]. Adoptive transfer experiments identified a role for CD4^+^ T cells in limiting the half-life of cells expressing DNA vaccine antigen [14]. Since immunity improved in Fas or FasL knockout (KO) mice, Fas-FasL signaling was implicated as a negative effector of DNA vaccine immunity. In their view, FasL+ CD4-suppressor cells had been destroying Fas+ antigen-presenting cells (APC) and knockout of Fas signaling preserved APC. We have repeated and extended the studies from the Letvin laboratory. Our studies address an old controversy about whether or not Fas-mediated apoptosis is beneficial for DNA vaccination [15]. One possible explanation for contradictory results is that most groups employed either pro-apoptotic [16,17] or anti-apoptotic molecules [18] but not both, in their specific delivery system (intramuscular injection or intradermal delivery by gene gun). We delivered DNA plasmids by intramuscular electroporation and explored both pro-apoptotic and anti-apoptotic molecules in regulating SIVmac Gag and HIV_BaL_ gp140 DNA vaccine immunity. Our approaches included Fas KO mice, antibodies against FasL, and the use of plasmids expressing Fas-pathway agonists or antagonists.

Finally, to expand the relevance of these studies outside HIV vaccination, we used two “adjuvant” plasmids, expressing Fas-associated death domain (FADD) or cellular FLICE-inhibitory protein (cFLIP), that promoted the clearance of chronic lymphocytic choriomeningitis virus (LCMV) infection. The death of activated T cells in chronic infections requires both the extrinsic Fas pathway (triggered by repeated T cell receptor stimulation) and the intrinsic “BCL2-regulated” pathway (triggered by a decline in growth factors) [19,20,21] reviewed by Strasser *et al.* [22]. Our studies showed that Fas pathway molecules are likely to have complex roles involving additional pathways in regulating DNA vaccine immunity.

## 2. Materials and Methods

### 2.1. Plasmids and Preparation

FasL MISSION shRNA (TRCN0000066640), the MISSION non-target shRNA control vector (SHC002, referred to as shRNA-non) and plasmid FasL-GFP were kind gifts from T.-C. Wu (Johns Hopkins School of Medicine). Plasmid IL-28B was a kind gift from Kenneth Bagley (Profectus Biosciences). It was created by replacing the yellow fluorescent protein (YFP) gene from pMAX-FP-Yellow (Amaxa Corp., Cologne, Germany) with a multiple cloning site using the Nhe-I and Bss-HII sites. The DNA sequence of mouse IL-28b was codon-optimized for expression in mouse cells by GenScript (Piscataway, NJ, USA). The optimized IL-28b sequence was sub-cloned into pMAX-PRO using the 5’ Kpn-1 and 3’ Xho-1 sites. The expression of IL-28 by this plasmid was verified using an IL-28 ELISA (Affymetrix/eBioscience) according to the Manufacturer’s instructions. All the other MISSION shRNA plasmids, including shRNA-Fas (TRCN0000012329), shRNA-FADD (TRCN0000012283) and shRNA-caspase 8 (TRCN0000231279) were ordered from Sigma-Aldrich (St. Louis, MO, USA). Plasmids pcDNA3.1-gp140 (HIV-1 BaL strain) and the empty pcDNA3.1 vector was obtained from Invitrogen (Grand Island, NY, USA). Plasmids pJ603-gag (the SIVmac gag gene encoding the Gag capsid protein p27), pJ603-Fas, pJ603-FADD and pJ603-cFLIP were ordered from DNA 2.0 (Menlo Park, CA, USA). The murine sequences for Fas, FADD and cFLIP were confirmed by sequence maps provided by DNA 2.0. All plasmids used for mouse immunization were prepared using an endotoxin-free plasmid kit from Qiagen (Valencia, CA, USA).

### 2.2. Peptides, Antibodies, and Diagnostic Kits for Flow Cytometry and Western Blots

The following reagents were obtained through the AIDS Research and Reference Reagent Program, Division of AIDS, NIAID, NIH: SIVmac 239 Gag (15-mer) Peptides-Complete Set, HIV-1 Consensus Subtype B Env (15-mer) Peptides-Complete Set, recombinant HIV-1BaL gp120, recombinant SIVmac251 Gag Pr55, HIV-1 gp120B sheep antiserum and mouse monoclonal antibody (mAb) to SIV p27. Rabbit polyclonal antibodies for FADD (AV30294) and FLIP (F9800) were ordered from Sigma-Aldrich. Rabbit polyclonal antibodies for Fas (sc-1023) and caspase-8 (AB1879) were ordered from Santa Cruz Biotechnology (Santa Cruz, CA, USA) and Millipore (Temecula, CA, USA) respectively. Monoclonal antibody for β-actin (A5441) was ordered from Sigma-Aldrich. Horseradish peroxidase (HRP) conjugated goat anti-rabbit IgG were ordered from Invitrogen (Grand Island, NY, USA). Recombinant Protein G was from Sigma-Aldrich. ECL Plus Western Blotting Detection System was from GE Healthcare (Pittsburgh, PA, USA). PE Annexin V Apoptosis Detection Kit, and Cytofix/Cytoperm Plus Fixation/Permeabilization kit were from BD Biosciences (San Jose, CA, USA). Anti-mouse Fas mAb Jo2 was also from BD Biosciences. Anti-Mouse/Rat Foxp3 staining Set PE and all the antibodies used for FACS staining were from eBiosciences (San Diego, CA, USA). Purified Armenian hamster IgG isotype (HTK888) and purified anti-mouse/rat CD178 (FasL) (MFL4) were from BioLegend (San Diego, CA, USA). APC-AL11 tetramer H-2D (b)/AAVKNWMTQTL, which was used to detect the dominant CD8^+^ T lymphocytes to SIVmac239 Gag [23], was obtained from NIH Tetramer Facility (Emory University).

### 2.3. Assays for Gene Expression and Apoptosis in Cell Culture

*In vitro* transfection reagent TurboFect was from Fermentas Inc. (Glen Burnie, MD, USA). For Western blot assay, plasmids pcDNA3.1-gp140, pJ603-gag, pJ603-FADD, pJ603-cFLIP, pJ603-Fas, shRNA-FADD and shRNA-Fas were transfected into L-929 cells. Cells were harvested 48h after transfection. Flow cytometry staining was also used for *in vitro* gene silencing assays. Plasmids FasL-GFP, shRNA-non, shRNA-FasL and pJ603-Fas were transfected into L-929 cells. Cells were harvested for surface antigen staining 48h later. For apoptosis assays, plasmids shRNA-non, shRNA-FADD and shRNA-caspase 8 were transfected into P815 cells. Here plasmid shRNA-non was used as a negative control to monitor the influence of plasmid transfection on cell apoptosis. Anti-mouse Fas mAb Jo2 (10 μg/mL) and Protein G (2 μg/mL) were added to cells 24h later to induce cell apoptosis. After 2h’s antibody incubation, cells were harvested for Annexin V staining.

### 2.4. *In Vivo* Immunizations

Animal protocol #0110017 was approved by the Institutional Animal Care and Use Committee of the University of Maryland. Seven- to eight-week-old female C57BL/6 mice and cognate Fas KO mice (The Jackson Laboratory, Bar Harbor, ME, USA) were anesthetized by i.p. injection of ketamine/xylazine (1:1 ratio). Skin overlying the tibialis anterior (TA) muscle was shaved and the animals were injected with plasmid DNA in 50 µL of sterile PBS. Co-administration of various gene plasmids involved mixing them before injection. Electroporation was modified from Widera *et al.* [24]. Two-needle array electrodes (5 mm gap, BTX, Holliston, MA, USA) were inserted into the muscle immediately after DNA delivery for electroporation. *In vivo* electroporation parameters were: 20 V/mm distance between the electrodes; 50 ms pulse length; 6 pulses; and 200 ms in between pulses, given by Electro Square PoratorTM ECM830 (Genetronics, Inc., San Diego, CA, USA). Depending on the experiment, one booster injection was carried out two or four weeks after the primary inoculation. Though it is now clear that TLR9-mediated recognition of plasmid DNA (unmethylated CpG motifs) and subsequent signaling are not essential for optimal DNA vaccination [4], we still added pcDNA3.1 or shRNA-non to make sure all the vaccinated mice received the same quantity of plasmids.

### 2.5. Cellular Immune Response Assays

Mice were euthanized at day 10 or day 36 after the last immunization. One million freshly prepared mouse splenocytes were used for tetramer staining and phenotype assays. APC-AL11 tetramer, CD8-FITC, CD44-PE and CD62L-PE-Cy7 were combined to stain. CD8^+^ T lymphocytes from empty vector-vaccinated mice were utilized as negative controls and exhibited less than 0.1% tetramer staining. For intracellular cytokine staining, 2 x 10^6^ splenocytes were seeded in 96-well plates and incubated overnight with peptide mixtures (0.5 μg/mL each single peptide) for SIVmac 239 Gag or HIV-1 Subtype B envelope in the presence of BD GolgiPlug (BD Biosciences). All the staining procedures were according to the protocol from the Cytofix/Cytoperm Plus Fixation/Permeabilization kit. For regulatory T (Treg) cell staining, 2 x 10^6^ splenocytes were seeded in 96-well plates and incubated overnight with peptide mixtures (0.5 μg/mL) for SIVmac 239 Gag or HIV-1 Subtype B envelope. All the staining procedures were according to the protocol from the anti-mouse/rat Foxp3 staining Set PE kit. Analyses were performed on a BD FACSCalibur flow cytometer with FlowJo 7.6.5 (Tree Star, Inc. Ashland, OR, USA).

### 2.6. Cell Proliferation Assays

Freshly prepared mouse splenocytes were suspended in prewarmed PBS/0.1%BSA at 2 x 10^6^ cells/mL. Five millimolar stock CFSE (Invitrogen) was added for a 2 μM final concentration. The reaction was incubated for 10 min at 37 °C. After quenching the staining by adding ice-cold cultured medium (RPMI-1640) on ice for 5 min, cells were washed twice with RPMI-1640 with 10% FCS. Labeled cells (2 x 10^6^) were plated into 24-well plates in 1 mL of medium and cultured for 3.5 days before cells were harvested for surface staining and subjected to flow cytometry.

### 2.7. Humoral Immune Response Assays

Serum anti-Gag and anti-gp140 antibody titers from immunized mice were measured by ELISA. 96-well plates (Costar, Corning, NY, USA) were coated overnight with 1 µg/mL recombinant SIV_mac251_ Pr55 Gag or HIV-1_Bal_ gp120 (NIH AIDS Research & Reference Reagent Program, Germantown, MD, USA) in 0.05 M carbonate-bicarbonate buffer (pH 9.6). After washing with PBST buffer (PBS containing 0.05% Tween-20), the plates were blocked with 5% nonfat dry milk in PBST buffer for 1 h at room temperature. Sera were then added in serial dilutions and incubated for 1 h at 37 °C. The plates were washed 6 times before HRP-conjugated goat anti-mouse IgG, IgG1 and IgG2a (Invitrogen, Frederick, MD, USA) were added for another 1 h incubation. The plates were washed 6 more times, developed with SureBlue™ TMB microwell peroxidase substrate (KPL, Gaithersburg, MD, USA), stopped with TMB stop solution, and analyzed at 450 nm with Wallac 1420 VICTOR2™ spectrophotometer (Perkin Elmer, Shelton, CT, USA). We now know that C57BL/6 mice lack IgG2a, and have IgG2c instead [25] so our ELISA results are lower than they would have been had we used anti-IgG2c.

### 2.8. LCMV Infections, DNA Electroporations and CMI Assays

C57BL/6 mice were used for all experiments with lymphocytic choriomeningitis virus (LCMV). Mice were electroporated with 2 µg plasmid (either a control plasmid pCDNA.3.1 or pJ603-FADD or pJ603-cFLIP) on day zero (d0) and then, on day 7, infected with either 2 × 10^5^ plaque-forming-unit (PFU) LCMV-Armstrong i.p. or 2 × 10^6^ PFU LCMV-Clone13 i.v.. Nine days later, mice were sacrificed and assessed for viremia and cell-mediated immunity. Cell-mediated immunity was assessed using splenocytes that were incubated with LCMV peptides followed by intracellular staining and flow cytometry as described [26]. LCMV peptides included H-2K^b^-GP34: AVYNFATM, H-2D^b^-GP33: KAVYNFATM, H-2D^b^-GP276: SGVENPGGYCL, and H-2D^b^-NP396: FQPQNGQFI. Viremia was assessed using a sensitive assay for infectious plaque forming units (PFU) in mouse serum as described [27]. Simple plaque assays can detect >100 PFU/mL of serum, whereas the sensitive two-step assay can detect virus in samples with as few as 10 PFU/mL of serum.

### 2.9. Statistical Analysis

Data were analyzed by GraphPad Prism 5. For group-to-group comparisons, the unpaired Student *t* test was employed. *P* < 0.05 was considered statistically significant. A multiple comparison analysis using the Tukey-Kramer method and one-way ANOVA has also been done and is tabulated in Table S1.

## 3. Results

### 3.1. Plasmid Expression and Pro- or Anti-Apoptotic Function were Confirmed in Cell Culture

It has been shown that Fas signaling negatively regulates DNA vaccine potency in studies with Fas or FasL KO mice [13,14]. We asked whether inhibiting Fas signaling would have a different effect on DNA vaccine efficacy. Before the *in vivo* studies, we first checked the function of several plasmids in cell culture. Western blot studies showed that the two model antigens HIV BaL envelope gp140 (140 kDa) and SIVmac Gag p27 (27 kDa) expressed well in L-929 cells after transfection (Figure S1A). The anti-apoptotic molecules cFLIP (55 kDa) and Fas (48 kDa) together with pro-apoptotic molecule FADD (27 kDa) also expressed the right-size protein in L-929 cells (Figure S1A). We detected endogenous signals for FADD, Fas in L-929 cells (Figure S1A). Little endogenous signal was detected for cFLIP. Compared to endogenous signals for FADD, Fas and caspase-8, weaker signals were detected by transfecting L-929 cells with plasmids shRNA-FADD, shRNA-Fas and shRNA-caspase-8.

For gene silencing studies, we also used flow cytometry staining to show that shRNA-FasL down-regulated both GFP and FasL expression when it was co-transfected with plasmid FasL-GFP into L-929 cells (Figure S1B). shRNA-Fas also down-regulated Fas expression when it was co-transfected with plasmid pJ603-Fas (Figure S1B). For apoptosis studies, we transfected plasmids into P815 cells, which had high Fas expression and could be driven to a high percentage of apoptosis by Fas crosslinking [16]. Here, we confirmed the strategy and further showed that shRNA-FADD and shRNA-caspase 8 could reduce Fas-induced apoptosis (Figure S1C). The percentage of background apoptosis in P815 cells without anti-Fas treatment was below 5% (data not shown). The flow data (Figure S1C) shows that caspase 8 shRNA reduced cell death. Our *in vitro* data, whether from Western blot, surface staining or apoptosis inhibition, all showed that our plasmids (including shRNA constructs) were functional.

### 3.2. Fas Signaling Had a Negative Influence on DNA Vaccine Potency

**Figure 1:**
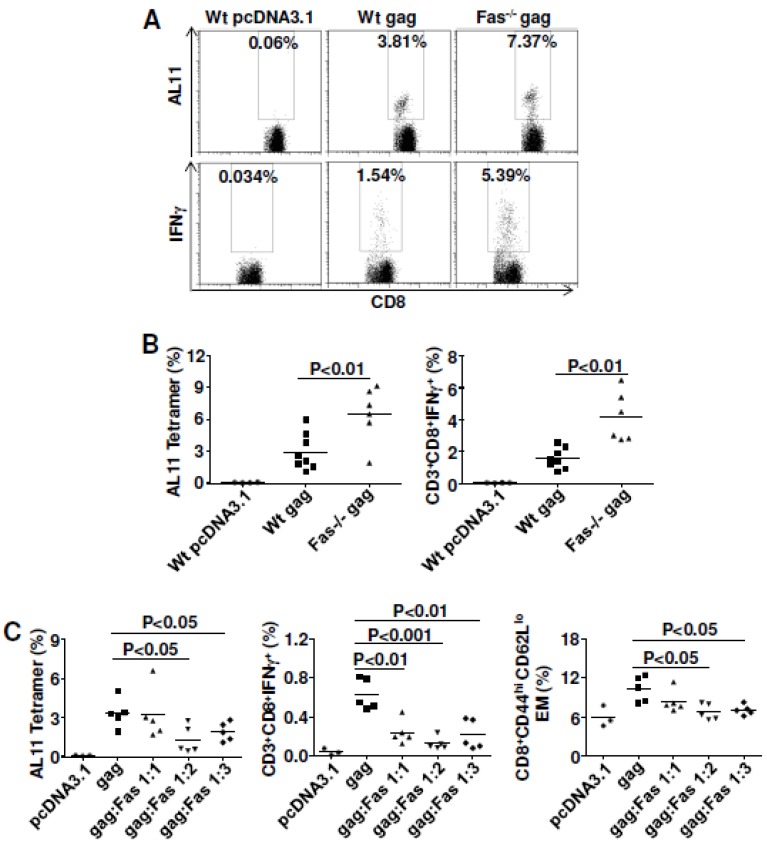
Fas gene-expression plays a negative role in regulating cell-mediated immune (CMI) responses induced by DNA vaccination. (**A** and **B**) CMI were significantly improved for both quantity and quality in Fas knock-out (KO) mice. Wild type C57BL/6 mice and their cognate KO mice were immunized with 10 μg plasmid pJ603-gag at day 0 and day 28. Mice were euthanized 10 days after the last immunization and splenocytes were prepared for CMI assay; (**A**) Representative plots were shown for tetramer staining (top) and intracellular cytokine staining (bottom); (**B**) Summary data for tetramer staining (left) and intracellular cytokine staining (right); (**C**) Overexpression of Fas seriously impaired cellular immune responses induced by DNA vaccination. Wild-type C57BL/6 mice were immunized with 2 μg plasmid pJ603-gag and different doses of plasmid pJ603-Fas at day 0 and day 14. Mice were euthanized 10 days after the last immunization and splenocytes were prepared for CMI assays; Data in **A** and **B** are collected from one single experiment (*n=*8 except for control group where *n=*4). Two Fas KO mice died before analysis; Data in **C** are representative of two independent experiments with similar results (*n=*5 except for control group where *n=*3). pcDNA3.1 was added to make sure all the vaccinated mice received the same quantity of plasmids.

We next tried to confirm earlier results from the Letvin group [13]. Wild type C57BL/6 mice or cognate Fas KO mice were immunized twice with SIVmac Gag plasmid. Mice were euthanized 10 days after the last immunization and splenocytes were prepared for cellular immune response assays. As published previously [13], and as shown here (Figure 1A,B), the quantity (tetramer for AL11 epitope) of cellular immune responses was greatly improved in Fas KO mice compared to wild type mice.

Whereas the Letvin group found no differences in the percentages of intracellular IFNγ between Fas KO mice and wild-type mice [13], we showed that the intracellular staining for IFNγ was significantly improved for Fas KO mice. This may be due to the fact that we performed tetramer analysis without antigen stimulation, whereas they did tetramer analysis after peptide stimulation, which has been shown to down-regulate TcR [28]. The vaccine effect we saw was more than two-fold improved (4.15 ± 0.63% of double positive CD8^+^IFNγ^+^ for KO mice compared to 1.56 ± 0.23% for wild-type mice). Importantly, we found that the function of CD4 T helper cells also greatly improved in Fas KO mice (Figure S2, 1.03 ± 0.16% of double positive CD4^+^IFNγ^+^ for KO mice compared to 0.54 ± 0.06% for wild type mice). These data indicated that Fas signaling played a negative role in regulating DNA vaccine potency.

To eliminate some possible unknown effect on vaccine efficacy from Fas KO mice, we carried out the Fas co-delivery experiment using wild-type C57BL/6 mice. We showed definitely that Fas co-delivery impaired cellular immune responses to SIVmac Gag with three different ratios between Gag plasmid and Fas plasmid (Figure 1C). The impaired responses might be due to the decreased effector memory cell populations (Figure 1C). The cell gating strategy for effector memory cell populations is shown in Fig. S3A. Besides the effects on secondary immune responses, Fas co-delivery also impaired the primary cellular immune responses (Figure S4). Rather than improving vaccine efficacy, overexpression of Fas impaired cellular immune responses after DNA vaccination. This observation was consistent with our studies with Fas KO mice, which indicated that Fas signaling negatively regulated DNA vaccine potency.

### 3.3. Inhibiting Fas or FasL (With shRNA or Antibody) Failed to Improve DNA Vaccine Immunity

**Figure 2:**
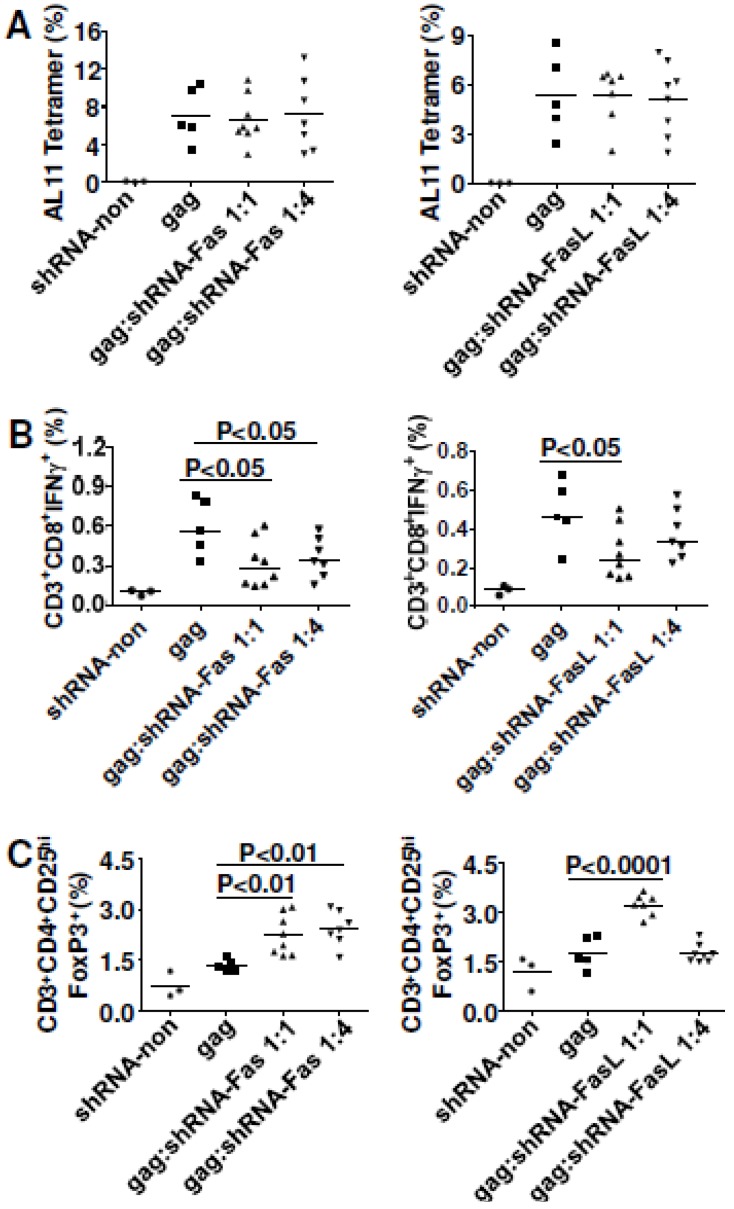
*In vivo* gene silencing of Fas and FasL did not improve CMI responses for SIVmac Gag. C57BL/6 mice were immunized with 2 μg plasmid pJ603-gag combined with 2 μg or 8 μg plasmids shRNA-Fas or shRNA-FasL at day 0 and day 28. Mice were euthanized 10 days after the last immunization and splenocytes were prepared for CMI assay. (**A**) Tetramer staining; (**B**) Intracellular cytokine staining; (**C**) Regulatory T cell assay; Data in **A**–**C** are representative of two independent experiments with similar results (*n=*5–8 except for control group where *n=*3). shRNA-non was added to make sure all the vaccinated mice received the same quantity of plasmids.

After demonstrating that Fas expression had negative effects on DNA vaccine potency, we checked whether *in vivo* silencing of Fas expression would be beneficial. We co-delivered shRNA-Fas or shRNA-FasL with Gag plasmid into normal C57BL/6 mice. Neither shRNA-Fas nor shRNA-FasL influenced the magnitude of cellular immune responses (Figure 2A). However, it was surprising that shRNA-Fas co-delivery seriously impaired the effector function of antigen-specific cytotoxic T lymphocytes with IFNγ as readout (Figure 2B). Similar impaired cellular immune responses were also detected with TNFα staining (Figure S5). Further studies showed that co-delivery of shRNA-Fas up-regulated Treg cells (Figure 2C). The cell gating strategy for Treg cells is shown in Figure S3B. For shRNA-FasL co-delivery, effector function of antigen-specific cytotoxic T lymphocytes was impaired when the ratio was 1:1 between Gag plasmid and shRNA-FasL while no change was seen when a 1:4 ratio was used (Figure 2B). shRNA-FasL co-delivery at 1:1 ratio also significantly up-regulated Treg cells (Figure 2C). Consistent with the effector function study, shRNA-FasL co-delivery at 1:4 ratio had no influence on Treg cells.

**Figure 3:**
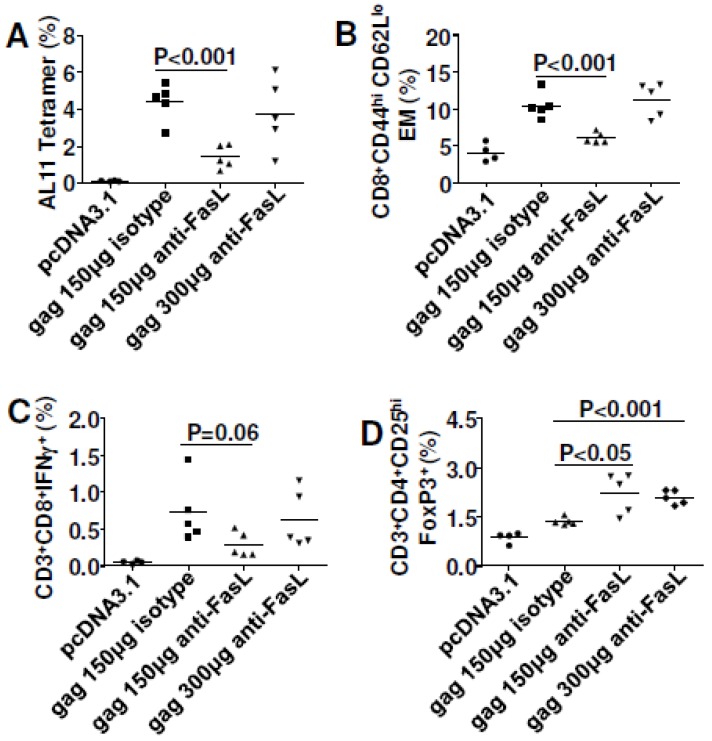
*In vivo* anti-FasL neutralization did not improve CMI responses for SIVmac Gag. C57BL/6 mice were immunized with 2 μg plasmid pJ603-gag at day 0 and day 28. Anti-FasL mAb was i.p. injected one day before the first immunization. After the first immunization, anti-FasL mAb was i.p. injected every four days until the end of the experiment. Mice were euthanized 10 days after the last immunization and splenocytes were prepared for cellular immune response assay. (**A**) Tetramer staining; (**B**) Effector memory cell assay; (**C**) Intracellular cytokine staining; (**D**) Regulatory T cell assay; Data in **A**–**D** are representative of two independent experiments with similar results (*n=*5 except for control group where *n=*4).

As the data from *in vivo* gene silencing of Fas and FasL were unexpected, we next used a different strategy for blocking the Fas signaling. C57BL/6 mice were injected i.p. with a FasL-neutralizing antibody one day before the first DNA vaccination; antibody treatment was repeated every four days until the end of the experiment. Our data showed that inoculating 150 μg anti-FasL impaired cellular immune responses in both quantity (Figure 3A) and quality (Figure 3C) and anti-FasL reduced effector memory CTL (Figure 3B). However, a 300 μg dose of anti-FasL had no influence on vaccine immunity, except for up-modulating Treg cells (Figure 3D).

### 3.4. Pro-Apoptotic FADD Enhanced Cellular Immune Responses to Gag

**Figure 4:**
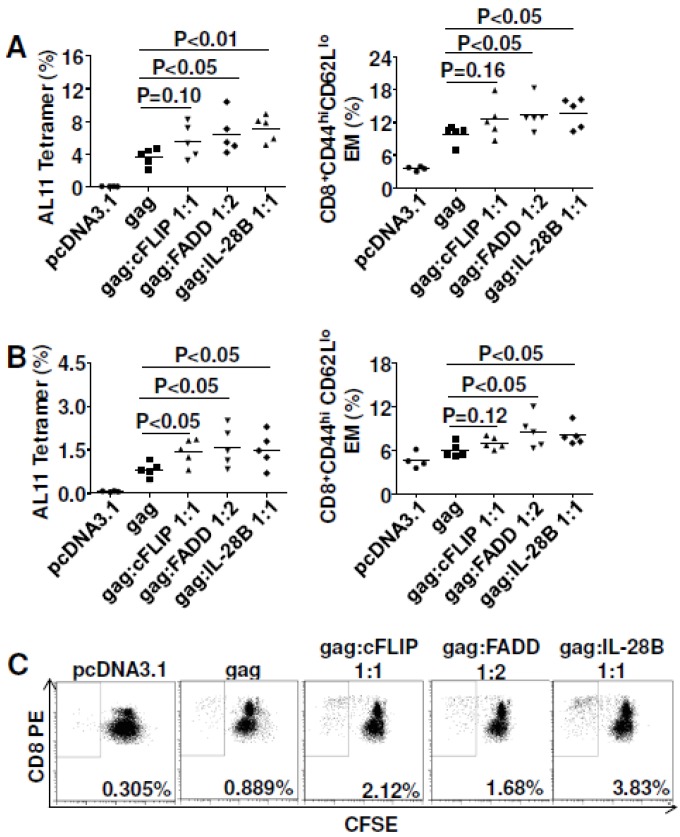
Fas-associated death domain (FADD) and IL-28B co-delivery improved cell-mediated immune (CMI) responses for SIVmac Gag. C57BL/6 mice were immunized with 2 μg plasmid pJ603-gag combined with 2 μg plasmid pJ603-cFLIP or plasmid IL-28B or 4 μg plasmid pJ603-FADD at day 0 and day 28. Mice were euthanized 10 (**A**) or 36 (**B** and **C**) days after the last immunization and splenocytes were prepared for cellular immune response assay; Data in **A–C** are representative of two independent experiments with similar results (*n=*5 except for control group where *n=*4). pcDNA3.1 was added to make sure all the vaccinated mice received the same quantity of plasmids.

Having shown that directly targeting Fas signaling (co-delivering shRNA-Fas and shRNA-FasL) impaired DNA vaccine immunity, we then checked the influence of molecules downstream of Fas signaling on vaccine efficacy. We tested a pair of molecules with opposite roles in the Fas pathway: the anti-apoptotic molecule cFLIP and the pro-apoptotic molecule FADD. We also tested IL-28B, a recently identified molecular adjuvant [8,29], as a positive control for our study. To our surprise, FADD significantly enhanced the magnitude of antigen-specific cellular immune responses (Figure 4A) and effector memory responses (Figure 4A) at 10 days after the last immunization. FADD and IL-28B had similar impacts on cellular immune responses, though the adjuvant effect depended on the plasmid ratio. There was no adjuvant effect at 1:1 ratio (data not shown). Electroporation of cFLIP was unable to significantly improve DNA vaccine potency at early stage (Figure 4A, left panel), but it did improve at late stage (Figure 4B, left panel).

To check whether the improved vaccine efficacy was sustained, we immunized mice with the same plasmid ratios but euthanized at 36 days after the last immunization. As was shown in Figure 4B, the adjuvant effect of FADD could be maintained for more than one month although the magnitude of cellular immune responses was lower compared to day 10. It has been reported that IL-28B can reduce Treg cells during DNA vaccination [8] and we checked Treg levels in our animals. Treg cells were upregulated significantly after FADD and IL-28B co-delivery at day 10 while at day 36 Treg were up for cFLIP and IL-28B co-delivery (Figure S6). Among the three molecules, IL-28B induced the highest levels of Treg cells. To explore the potential mechanism of the adjuvant effect, splenocytes from mice euthanized at day 36 were combined together within each group and stained with CFSE. Cells stained with CFSE were cultured for three and a half days with Gag peptide pools, and then collected for flow cytometry. As was shown in Figure 4C, IL-28B was most effective in enhancing the proliferative capacity of Gag-specific CTL. cFLIP and FADD also enhanced cell proliferation, though to a lesser degree than IL-28B. None of these three molecules influenced proliferation of antigen-specific Th1 cells (data not shown). Besides cellular immune responses, we also checked humoral immune responses and found that none significantly enhanced Gag-specific total IgG and IgG2a (Figure S7).

### 3.5. Both cFLIP and FADD Improved Vaccine Efficacy to HIV_BaL_ gp140

**Figure 5:**
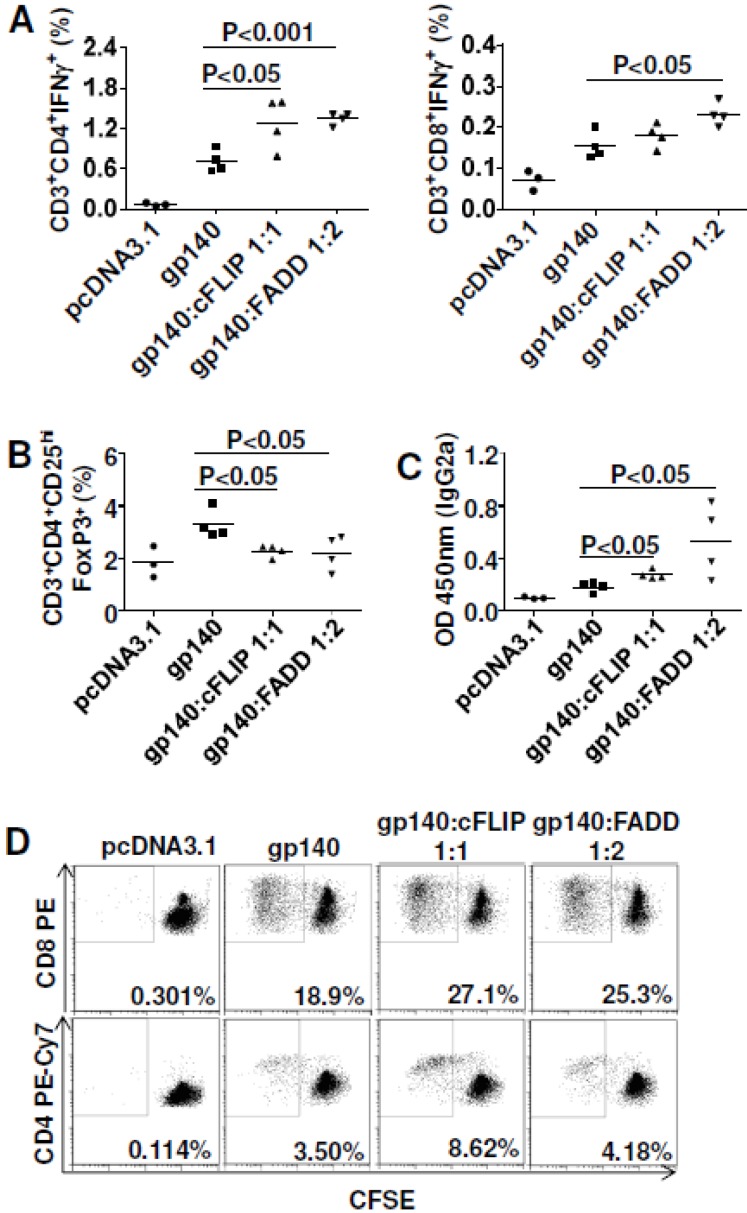
Cellular FLICE-inhibitory protein (cFLIP; FLICE is FADD-like IL-1beta converting enzyme) and FADD co-delivery improved both cellular immune responses and humoral immune responses for HIV gp140. C57BL/6 mice were immunized with 10 μg plasmid pcDNA3.1-gp140 combined with 10 μg plasmid pJ603-cFLIP or 20 μg plasmid pJ603-FADD at day 0 and day 28. Mice were euthanized 10 days after the last immunization and splenocytes were prepared for cellular immune response assay. Blood was collected during euthanasia to prepare sera for ELISA. Sera were diluted 100-fold for antibody titration. (**A**) Intracellular cytokine staining; (**B**) Regulatory T cell assay; (**C**) Humoral immune responses for IgG2a; (**D**) Cell proliferation assay for CTLs (top) and Th cells (bottom); Data in **A**–**D** are representative of two independent experiments with similar results (*n=*4 except for control group where *n=*3). pcDNA3.1 was added to make sure all the vaccinated mice received the same quantity of plasmids.

To check whether the effects for cFLIP and FADD were antigen-dependent, we switched the model antigen from SIVmac Gag to HIV_Bal_ gp140. Consequently, both cFLIP and FADD enhanced CMI to gp140, especially Th1 cells (Figure 5A).

Consistent with other reports, our data showed that cellular immune responses to gp140 were Th1 dominant [30,31]. In contrast to Gag plasmid immunization, both cFLIP and FADD significantly down-regulated Treg cells after gp140 immunization (Figure 5B). As both neutralizing and non-neutralizing antibody responses to HIV envelope are important for vaccine protection from HIV [32,33], we also measured humoral immune responses to gp140. Neither cFLIP nor FADD affected total IgG or IgG1 to gp140 (Figure S8), but they significantly improved IgG2a responses indicating that both cFLIP and FADD were Th1-polarized adjuvants for DNA vaccines (Figure 5C). We also found that both cFLIP and FADD enhanced proliferation of gp140-specific CTL and Th1 cells (Figure 5D). Thus, cFLIP and FADD are both adjuvants for gp140 vaccination, and their adjuvant effects were not antigen-dependent.

### 3.6. In Vivo Gene Silencing of Caspase 8 Enhanced DNA Vaccine Potency

**Figure 6:**
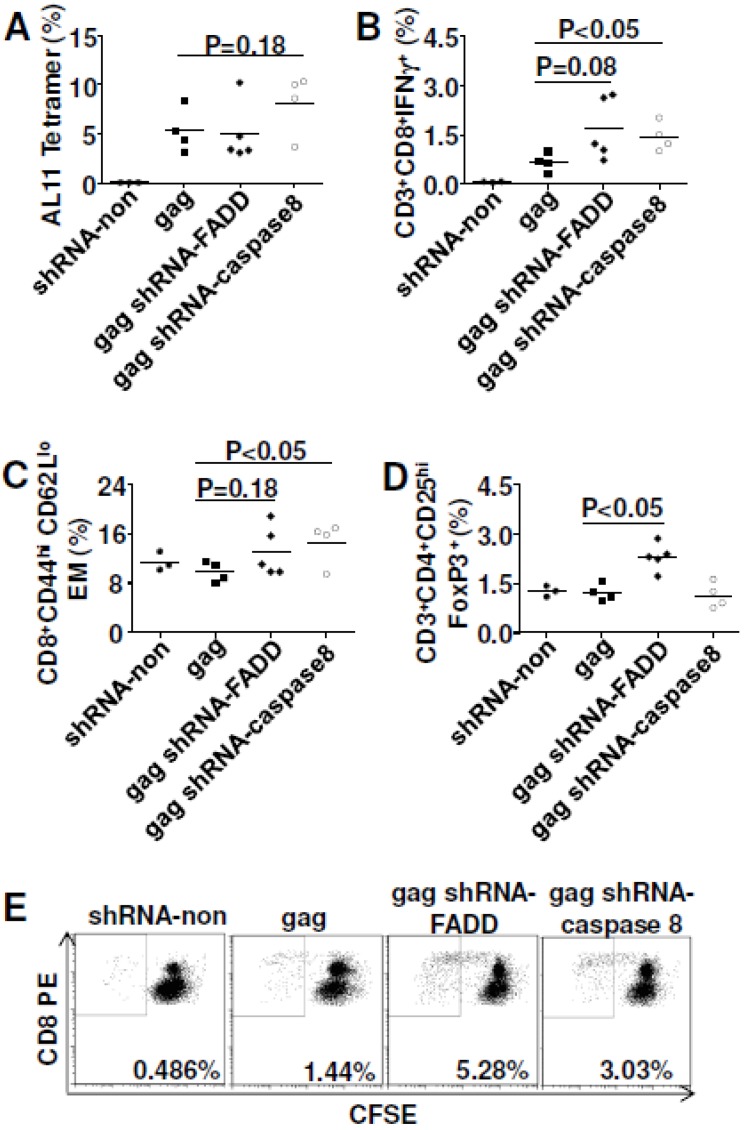
*In vivo* gene silencing of caspase 8 improved CMI responses for SIVmac Gag. C57BL/6 mice were immunized with 2 μg plasmid pJ603-gag combined with 2 μg plasmid shRNA-FADD or shRNA-caspase 8 at day 0 and day 28. Mice were euthanized 10 days after the last immunization and splenocytes were prepared for CMI assay. (**A**) Tetramer staining; (**B**) Intracellular cytokine staining; (**C**) Effector memory cell assay; (**D**) Regulatory T cell assay; (**E**) Cell proliferation assay; Data in **A**–**E** are representative of two independent experiments with similar results (*n=*4–5 except for control group where *n=*3). shRNA-non was added to make sure all the vaccinated mice received the same quantity of plasmids.

As we already showed, FADD co-delivery with SIVmac Gag was able to improve vaccine efficacy; so now we asked whether *in vivo* silencing of FADD would impair DNA vaccine potency. At the same time, we also checked the effect of silencing caspase 8, an important initiator caspase for Fas signaling [22], on Gag-specific vaccine immunity. *In vivo* silencing of FADD and caspase 8 did not influence the magnitude of Gag-specific cellular immune responses (Figure 6A), but did enhance the effector function, especially for caspase 8 silencing (Figure 6B). In addition, gene silencing of caspase 8 significantly enhanced effector memory CTL to Gag (Figure 6C). Gene silencing of FADD had no effect on effector memory (*p* = 0.18). To explore the potential mechanism, Treg cells were also analyzed. We found that gene silencing of caspase 8 failed to down-regulate Treg while gene silencing of FADD up-regulated Treg (*p*<0.05) (Figure 6D), which indicated that improved cellular immune responses do not always correlate with down-regulated Treg cells. We further checked cell proliferation ability after *in vivo* silencing of FADD and caspase 8. As observed for cFLIP or FADD co-delivery, gene silencing of FADD and caspase 8 both greatly enhanced the proliferation of Gag-specific CTL (Figure 6E). There was no influence on the proliferation of Gag-specific Th1 cells (data not shown).

### 3.7. cFLIP and FADD Vaccination Helped Clear Chronic Virus LCMV-Clone13 in Mice

Since we showed that both cFLIP and FADD were capable of improving cellular immune responses to SIVmac Gag and HIVBal gp140, we asked whether they could influence virus infection *in vivo*. We injected C57BL/6 mice with plasmid cFLIP or FADD followed by electroporation. Seven days later, mice were infected with lymphocytic choriomeningitis virus (LCMV). Nine days after infection, mice were sacrificed and assessed for viremia and cell-mediated immunity.

The rationale for this protocol was that the LCMV chronic infection model depends on the failure of CMI in which the chronic virus (LCMV-Clone13) causes T cell exhaustion by inducing regulatory molecules like PD1 [34] or Tim-3 [26]. We pre-injected mice with FADD or cFLIP plasmids to improve the immune environment during infection, thus facilitating viral clearance.

**Figure 7:**
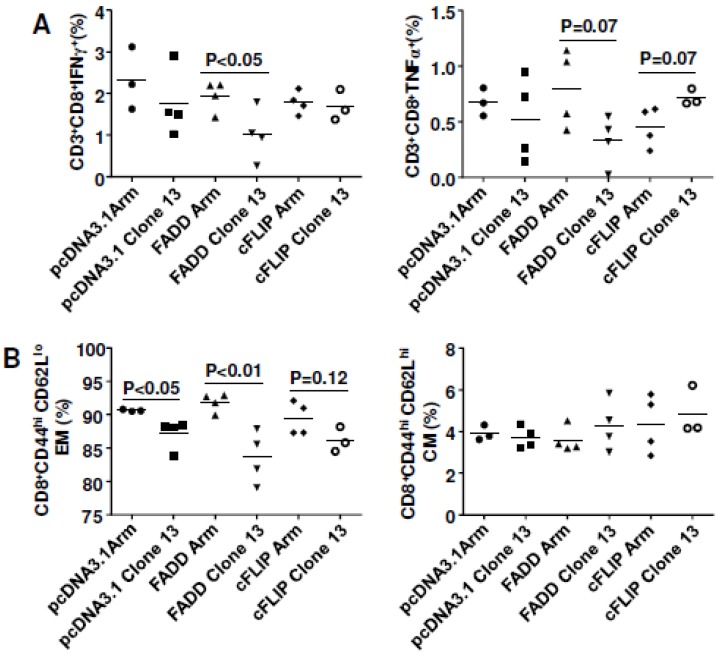
cFLIP maintained cellular immune responses in chronic lymphocytic choriomeningitis virus (LCMV) infection. Mice were electroporated with 2 µg plasmids of pcDNA.3.1, FADD or cFLIP on day zero (d0). On day 7, mice were infected with either 2 x 10^5^ plaque-forming-units (PFU) LCMV-Armstrong i.p. or 2 x 10^6^ PFU LCMV-Clone13 i.v.. Nine days later, mice were sacrificed and assessed for cell-mediated immunity. (**A**) Intracellular cytokine staining; (**B**) Memory cell responses.

**Figure 8:**
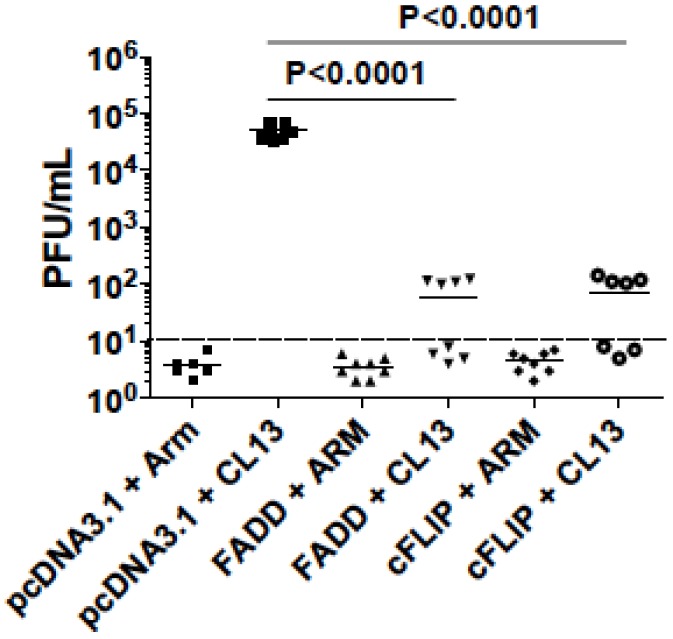
LCMV-CL13 persistence is less frequent in mice given FADD or cFLIP plasmids. On day0, C57BL/6 mice were electroporated with either pcDNA3.1 (control) or plasmids expressing FADD, or cFLIP. On day7, mice were infected with LCMV-Armstrong (Arm) or LCMV-Clone 13 (CL13), and on day 16, mice were sacrificed and assessed for CMI and viremia as described in Methods. The limit of detection for simple plaque assays is 10^2^ PFU/mL and the limit of detection for the 2-step assays is 10 PFU/mL, so mice with less than 10 PFU/mL are considered to have “cleared” the virus. Data were representative of two repeated experiments.

As a result, inoculation of LCMV-CL13-infected mice with cFLIP plasmid resulted in higher virus-specific CMI, whereas co-inoculation with FADD plasmid did not significantly affect CMI (Figure 7A). Further analyses showed that cFLIP could maintain effector memory cells in chronic infection while both pcDNA3.1 and FADD failed (Figure 7B). Surprisingly, even though chronic infection still deleted CD8 T cells (Figure S9), almost half of the mice cleared chronic virus LCMV-Clone 13 when mice were injected with either cFLIP or FADD plasmid (Figure 8). All the mice injected with pcDNA3.1 failed to clear chronic LCMV-Clone 13.

## 4. Discussion

Understanding host mechanisms that modulate immune responses to DNA immunization may help improve the low immunogenicity of DNA vaccines in clinical trials. Apoptosis is one of these mechanisms and has long been considered to influence vaccine efficacy [16,17,18,35,36,37]. However, seemingly contradictory reports over the last two decades have confused this area [15]. We conducted a systematic study employing several pro-apoptotic and anti-apoptotic molecules in the Fas extrinsic apoptosis pathway [38] and tested their effects on DNA vaccine potency (Table 1).

A complex relationship exists between Fas pathway molecules and vaccine efficacy. Neither inhibiting apoptosis (cFLIP and shRNA-FasL co-delivery, neutralizing FasL by mAb) nor improving apoptosis (FADD co-delivery) absolutely enhanced or decreased DNA vaccine potency in our studies (except for pro-apoptotic molecule Fas that in all situations down-regulated cellular immune responses, Figure 1C), which challenges existing models about the role for apoptosis in DNA vaccine potency [15]. We showed that the influence on DNA vaccine immunity differed depending on the molecule used, and also depended on the ratio between antigen and apoptosis modifier (Table 1). Anti-apoptotic molecule cFLIP acted as an adjuvant to enhance DNA vaccine potency when the ratio between Gag and cFLIP plasmids was 1:1 (no influence at 1:2 ratio). Pro-apoptotic molecule FADD showed adjuvant effect when 1:2 ratio of Gag to FADD plasmids was used rather than at the 1:1 ratio. The shRNA-FasL is another dose-dependent example where a 1:1 ratio between Gag and shRNA-FasL plasmids impaired cellular immune responses to Gag while there was no change for the 1:4 ratio. Considering that Dr. Wu’s group [39] reported enhanced DNA vaccine potency at a 1:9 ratio between HPV-E7 and shRNA-FasL (the same shRNA plasmid as ours), we reasoned that vaccine enhancement *in vivo* depended on the degree of FasL silencing. At a lower silencing level (1:1 ratio in our study), vaccine efficacy was impaired; at a moderate silencing level (1:4 ratio in our study), there was no influence on vaccine efficacy; at a higher level (1:9 ratio in Dr. Wu’s study), vaccine efficacy was enhanced. This pattern was also reflected by our antibody blocking experiment (Figure 3) where 150–300 μg of FasL neutralizing mAb had little to no effect on vaccine efficacy. Five hundred micrograms of this antibody used by others [40] enhanced B cell responses to *T cruzi*. When FasL was silenced completely *in vivo*, as with the FasL KO model, vaccine efficacy was definitely improved [14]. Interestingly, this *in vivo* gene-silencing model also works for Fas; we showed both 1:1 and 1:4 ratios between Gag and shRNA-Fas impaired vaccine efficacy. Meanwhile, our current study (Figure 1A,B) and work from Dr. Letvin’s group [13] both showed that DNA vaccine immunity was significantly improved in Fas KO mice compared to the wild type mice. Further studies are needed to investigate why moderate *in vivo* silencing of Fas or FasL impairs DNA vaccine potency.

One important finding of our studies is that both FADD and cFLIP were able to enhance DNA vaccine potency. They acted as molecular adjuvants for DNA immunization for both SIVmac Gag and HIV_Bal_ gp140. As for the potential mechanisms explaining the adjuvant effects, we found that Gag-specific effector memory CD8^+^ T cells were greatly improved with these adjuvants (Figure 4A,B). Furthermore, antigen-specific CD8^+^ T cell proliferation was observed (Figure 4C). Importantly, enhanced cell proliferation was also observed for HIV_BaL_ gp140 antigen (Figure 5D). Interestingly, *in vivo* gene silencing of caspase 8 also induced higher effector memory CD8^+^ T cells (Figure 6C) and cell proliferation (Figure 6E). Since caspase 8 has roles in FasL-triggered cell death, in inflammasome priming [41] and in suppressing the endogenous necrotic cell death pathway, necroptosis [42], its precise function in our studies remains unresolved. Our findings imply that improving antigen-specific effector memory CD8^+^ T cells and their proliferation may be good options for enhancing DNA vaccine potency [43].

**Table 1:** Modulation of cellular immune responses in mice after DNA vaccination by co-delivering Fas-pathway molecules ^1^.

Antigen	Plasmid-Expressed Molecules (Pro- or Anti-Apoptotic)	Ratio	Cellular Immune Responses (CMI)	
			AL11 Tetramer	CD3CD8 IFNγ
SIVmac Gag	Fas (pro-)	1:1	no change	decrease
		1:2,1:3	decrease	decrease
	shRNA-Fas (anti-)	1:1,1:4	no change	decrease
	shRNA-FasL (anti-)	1:1	no change	decrease
		1:4	no change	no change
	cFLIP ((anti-)	1:1	increase	increase
	FADD (pro-)	1:2	increase	increase
	shRNA-FADD (anti-)	1:1	no change	increase
	shRNA-caspase 8 (anti-)	1:1	increase	increase
HIV_BaL_ gp140	cFLIP (anti-)	1:1	NA	increase
	FADD (pro-)	1:2	NA	increase

^1^ C57 BL/6J mice were immunized with SIVmac Gag plasmid or HIV_BaL_ gp140 plasmid and corresponding pro- or anti-apoptotic molecules. One booster injection was followed 4 weeks later (except for Fas co-delivery with 2 weeks interval). Splenocytes were prepared 10 days after the last immunization. Cellular immune responses were analyzed by AL11 tetramer staining and intracellular cytokine staining for IFNγ. NA means no analysis of CMI. No change in the CMI after plasmid electroporation, with respect to control or empty plasmid is designated no change, whereas increase or decrease in CMI with respect to control plasmids is designated as increase or decrease.

It was interesting that co-delivery of cFLIP or FADD up-regulated Treg cells after Gag immunization (Figure S4) but Treg cells were significantly decreased when the antigen was gp140 (Figure 5B). The reasons for the antigen-dependent differences in Treg cells are not clear. However, the ability to down-regulate Treg cells for cFLIP or FADD with gp140 antigen may benefit HIV vaccine design considering the deleterious effects of Treg cells in HIV infection [44,45]. It was reported that Treg cells can restrict memory CD8^+^ T cell responses [46] and induce apoptosis of effector CD4^+^ T cells [47]. Using IL-28B as a positive control, we showed that FADD worked similarly to enhance DNA vaccine immunity to Gag though IL-28B was better for improving cell proliferative ability (Figure 4C, 3.83% compared to 1.68%). On the other hand, both FADD and IL-28B significantly up-regulated Treg cells, with IL-28B causing higher Treg induction (Figure S4). Our observation differed from a previous report [8] showing that IL-28B reduced Treg cells during DNA vaccination. This may reflect differences in the IL-28B plasmid vector backbone (ours was a pMAX-Pro vector described in Methods and theirs had a pVAX1 backbone [8]).

An important reason for the controversial reports about the role of apoptosis in regulating vaccine immunity is the lack of evidence about cell fate, since that is usually extrapolated from *in vitro* analysis [36,37] and may not reflect cell fates *in vivo*. The problem is that it is difficult to track down the cell fate *in vivo* after delivering shRNA plasmids to knock down specific genes, unless the plasmids express substantial reporter gene (e.g., luciferase) activity [39]. In our study, we did check the surface expression of Fas and FasL (by FACS staining) on lymphocytes from mouse spleen and found no significant changes after electroporating Fas pathway molecules (data not shown). Since we had difficulty assessing cell fates *in vivo* after co-delivering cFLIP and FADD (or the shRNA plasmids, such as shRNA-FADD), we are not sure whether the observed adjuvant effects are induced by prolonged lifespan for dendritic cells (mediated by cFLIP, shRNA-FADD and shRNA-caspase 8) or increased antigen-load in apoptotic bodies (mediated by FADD) [15]. We do not exclude the possibility that either cFLIP or FADD (or both) enhanced cellular immune responses using a mechanism other than apoptosis, especially considering the fact that Fas signaling is also involved in non-apoptotic processes [22], including cellular activation, differentiation, and proliferation. For example, soluble FasL is known to promote trafficking of monocytes and neutrophils [48], so our experiments with anti-FasL could have had their primary effects on trafficking. Besides cell fate analysis, in the future, it may be interesting to investigate whether cFLIP and FADD co-delivery affects the innate immunity which may contribute to the improved adaptive immune responses in the current study as all vaccine adjuvants appear to stimulate components of the innate immune system [10,49].

Another important finding in our studies is that Fas co-delivery impaired cellular immune responses to Gag (Figure 1C)*,* which corroborates the Fas knock-down data from our laboratory (Figure 1A,B) and from the Letvin laboratory [13], but contradicts older data [16]. The difference might be explained by the fact that the older report used Balb/c rather than C57BL/6 mice and DNA inoculation rather than electroporation. The strongest evidence supports the hypothesis that Fas negatively affects DNA vaccine potency.

Plasmids shown to boost immunity with HIV vaccination also influenced a model of chronic infection based on lymphocytic choriomeningitis virus. A variant of LCMV-Armstrong (ARM) named LCMV-Clone13 (CL13), which differs by five nucleotides, or two amino acids, from its parental strain [50,51], fails to elicit the vigorous cell-mediated immunity elicited by the acutely-infecting ARM, and consequently persists longer in the mouse. We attempted to boost cell-mediated immunity to CL13 by electroporating mice with plasmids expressing Fas-associated death domain (FADD) or cellular FLICE-inhibitory protein (cFLIP) known to boost cell-mediated immunity to SIV gag. We found that overexpression of cFLIP could maintain effector memory cells in chronic LCMV infection, but we saw no effect with the FADD plasmid (Figure 7). However, both cFLIP and FADD overexpression hastened viral clearance from the CL13 (persistent variant)-infected mice (Figure 8). Although Arm virus is cleared in 100% of mice by day 9 after infection, all mice infected with CL13 fail to clear virus. Half of the CL13-infected mice given FADD or cFLIP plasmids, but not empty vectors had succeeded in clearing virus by day 9.

The modest effects of FADD and cFLIP plasmids on CMI are seemingly discordant with their ability to promote virus clearance. Since neither plasmid could prevent the depletion of CD8 T cells during chronic infection, we surmise that viral clearance needs only slight immune capacity, not entirely dependent on the preservation of antigen-presenting cells as postulated by the Letvin group [14]. We speculate that the mechanism of viral clearance is an increase in cross-presentation by antigen-presenting cells in the case of FADD expression. In the case of cFLIP expression, preservation of antigen-presenting cells was a more likely mechanism to promote viral clearance. In both cases we believe better antigen presentation accounted for increased CMI and virus clearance. Since cellular immune responses were not significantly influenced, we speculate that cFLIP and FADD may modulate the innate immunity to fight the infection, as it has been reported that blocking type I interferon helps control chronic LCMV infection [52,53]. Rafi Ahmed’s lab has published an assessment of the contribution of death ligand PD-L1 and Tregs to CMI and viral clearance with Treg depletion improving CMI, but both PD-L1 and Treg depletion were needed to reduce viral loads [50]. Our future studies will examine the balance of Treg and PD-L1 in FADD or cFLIP-treated mice.

**Figure 9:**
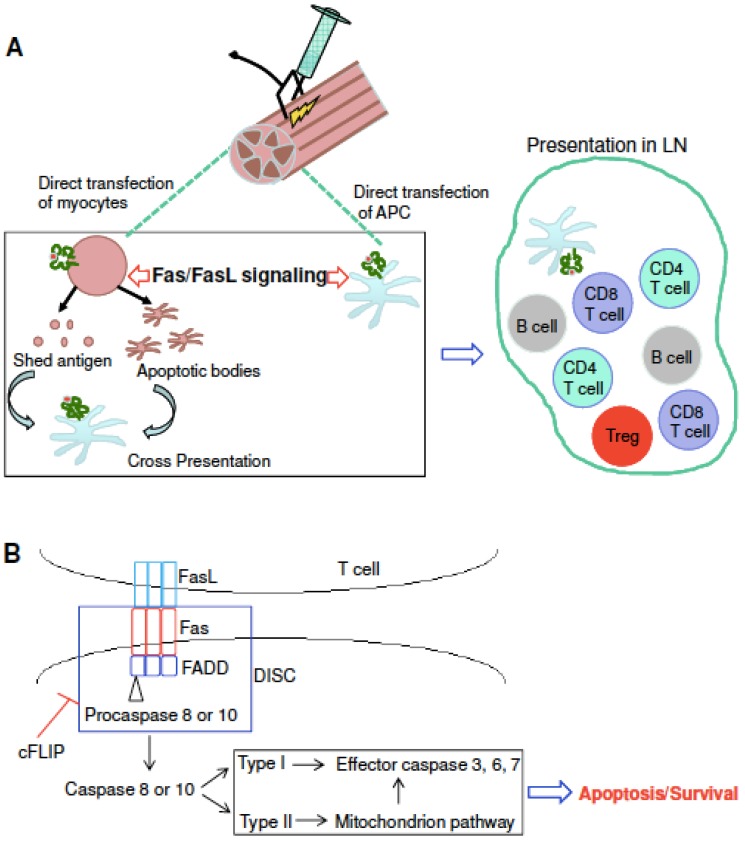
The fate of plasmids and their expressed antigens after electroporation (**A**); The Fas/FasL signaling pathway and some of the key molecules involved (**B**).

The potential mechanisms for modulating SIV/HIV DNA vaccine potency by Fas-FasL signaling are summarized using a cartoon model (Figure 9). Figure 9A shows how DNA vaccines induce both cellular and humoral immune responses through intramuscular injection followed by electroporation. It is now accepted that both myocytes and resident antigen presenting cells (APCs) are transfected with injected plasmids [54]. Therefore, strategies to protect the APCs would be expected to improve DNA vaccine potency. Indeed, by injecting mouse muscle with a plasmid expressing luciferase plus a dominant epitope from SIV Gag, Greenland *et al.* [13] detected persistent, high-level luciferase expression in Fas-deficient mice compared to decreased, low-level luciferase in wild type mice. Importantly, the author observed a persistent and potent immune response in Fas-/- animals in association with persistent antigen expression, which indicates that persistent immune responses can be generated if Fas-mediated antigen clearance is avoided. We successfully repeated their experiments using a plasmid expressing SIV Gag and showed that both quantity (tetramer for AL11 epitope) and quality (intracellular cytokine staining for IFNγ) of cellular immune responses were significantly improved in Fas KO mice compared to wild type mice (Figure 1A,B). In addition, we showed that Fas overexpression *in vivo* impaired vaccine potency (Figure 1C). Based on these data, we predict that Fas-mediated apoptosis of myocytes and APCs impairs the antigen pools, resulting in weak vaccine potency in wild type mice compared to Fas KO mice.

Both co-delivery of cFLIP *in vivo* and silencing of caspase 8 *in vivo* block Fas signaling (Figure 9B) and prevent the loss of cell-death-mediated loss of antigen pools, which results in improved vaccine potency (Figure 4 and Figure 5) and shRNA-caspase 8 effects (Figure 6). It is unexpected that overexpression of FADD (Figure 4 and Figure 5) improved vaccine potency. Cell proliferation experiments by CFSE staining showed that co-delivering FADD induced proliferation of antigen-specific lymphocytes, especially CD8+ T cells. Thus, rather than inducing apoptosis, co-delivering FADD activates proliferation, possibly through NF-κB signaling. Indeed, many reports have shown that not only FADD [55,56,57], but also Fas signaling [22,58] can induce proliferation rather than apoptosis. Considering these new findings, we predict that co-delivering shRNA-Fas or shRNA-FasL *in vivo* may interfere with proliferation signaling, resulting in impaired immunity in our system (Figure 2). Similarly, neutralization of FasL *in vivo* may also disturb some proliferation signaling. It should be remembered that there are many different proliferative (NF-κB, MAPK) and apoptotic (Fas and TRAIL) signaling pathways and their cross-talk could affect the final outcome. Future work may need to check these signaling pathways to confirm our proposed mechanism.

Taken together, we have identified two new molecular adjuvants cFLIP and FADD, both of which can improve cellular immune responses to SIVmac Gag and HIV_Bal_ gp140 after DNA vaccination by enhancing antigen-specific effector memory cells and promoting the proliferation of antigen-specific CTL. cFLIP and FADD also promote the proliferation of antigen-specific Th1 cells to gp140. In addition, we find that *in vivo* gene silencing of caspase 8 by shRNA also improves cellular immune responses to SIVmac Gag. To our knowledge, we are the first to show that cFLIP and FADD can significantly down-regulate Treg cells to HIV_Bal_ gp140. We are also the first to show that directly targeting Fas and FasL *in vivo* might impair cellular immune responses after DNA vaccination, whereas targeting downstream molecules in the Fas signaling pathway significantly enhanced DNA vaccine potency. Considering the recent failure of IL-12 and IL-15 as cytokine adjuvants in a human study [59], it will be important to have new adjuvants in the pipeline. Further investigations into the function of cFLIP and FADD, their role in SIV/HIV protection in monkey or humanized mouse models are needed to promote this process. It will also be important to investigate whether cFLIP and FADD can enhance anti-tumor immunity considering that they have the potential to down-regulate Treg cells, which constitute a major obstacle for tumor immunotherapy [60].

## Supplementary Files

Supplementary File 1
