# Supplementary material for: Modulation of SIV and HIV DNA Vaccine Immunity by Fas-FasL Signaling
Source: Viruses. 2015 Mar 23;7(3):1429–53. doi: 10.3390/v7031429 (PMC4379579; doi:10.3390/v7031429)
Supplement: Supplementary File 1 [file viruses-07-01429-s001.pdf]

Supplementary Materials

Modulation of SIV and HIV DNA Vaccine Immunity by Fas-FasL Signaling

Jiabin Yan, Juan Carlos Zapata, Charles David Pauza and Maria S. Salvato

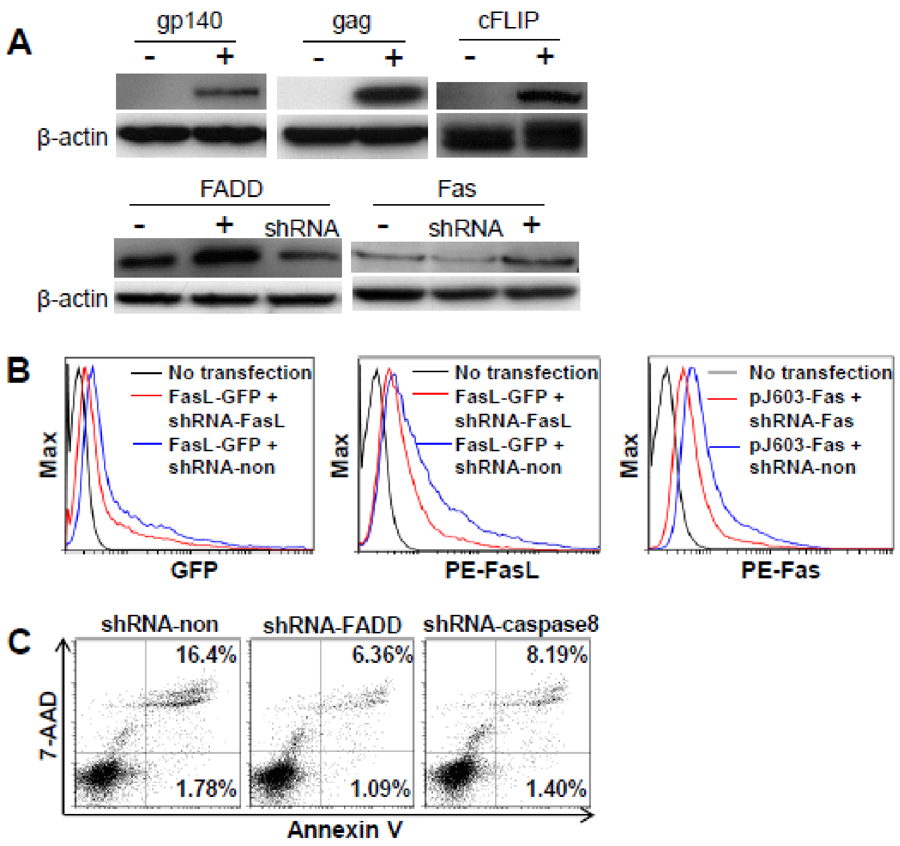

Figure S1. Evidence for gene expression and function of plasmids in cell culture. (**A**) Western blot assay. Plasmids pcDNA3.1-gp140, pJ603-gag, pJ603-cFLIP, pJ603-FADD, pJ603-Fas, shRNA-FADD, and shRNA-Fas were transfected into L-929 cells. Cell lysates were prepared 48h after transfection. HIV-1 gp120B sheep antiserum, mouse monoclonal antibody (mAb) to SIV p27, polyclonal antibodies for murine cFLIP, FADD, and Fas were used to detect the corresponding protein in cell lysates; (**B**) *In vitro* gene silencing assay. Plasmids FasL-GFP, shRNA-non, shRNA-Fas, shRNA-FasL and pJ603-Fas were transfected into L-929 cells. Cells were harvested 48h after transfection. Cell surface expression of GFP, Fas and FasL was analyzed by flow cytometry; (**C**) *In vitro* apoptosis assay. Plasmids shRNA-non, shRNA-FADD and shRNA-caspase 8 were transfected into P815 cells. 24h later, anti-Fas monoclonal antibody (Jo2 clone) and Protein G were added to incubate for 2h before apoptosis analysis by Annexin V staining. Both shRNA-FADD and shRNA-caspase 8 transfection reduced apoptosis induced by Fas antibody crosslinking. Data in (A) to (C) are representative of two independent experiments with similar results.

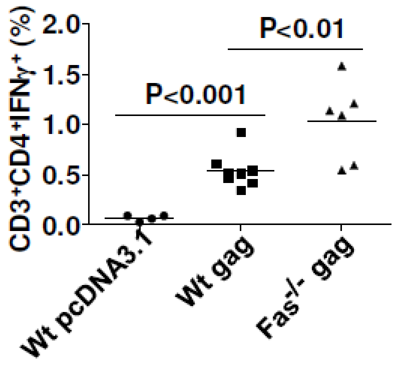

Figure S2. Fas signaling impaired the function of CD4 T helper cells induced by DNA vaccination. Wild type C57BL/6 mice and their cognate KO mice were immunized with 10 μg plasmid pJ603-gag at day 0 and day 28. Mice were euthanized 10 days after the last immunization and splenocytes were prepared for cell-mediated immune (CMI) response assay. Data for Fas KO + pcDNA3.1 control was similar to the wild-type control data.

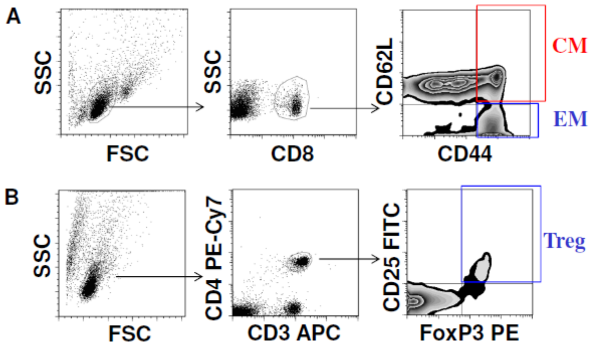

Figure S3. Cell gating strategies for memory cells and regulatory T (Treg) cells. (**A**) Memory CD8+ cells were gated on lymphocytes population from mouse splenocytes. Effector memory cells (EM) are defined as $CD44^{hi}CD62L^{lo}$ population while central memory cells (CM) are defined as $CD44^{hi}CD62L^{hi}$ population; (**B**) Treg cells are defined as $CD3^{+}CD4^{+}CD25^{hi}FoxP3^{+}$.

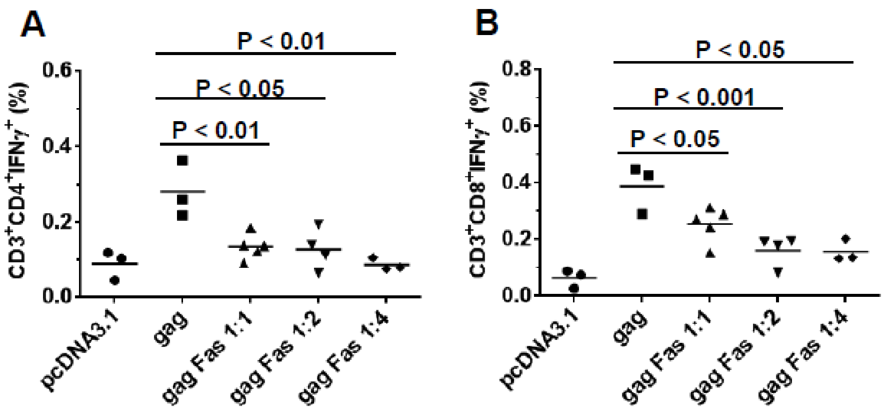

Figure S4. Fas overexpression impaired primary cell-mediated immune CMI responses induced by DNA vaccination. Wild type C57BL/6 mice were immunized with 10 μg plasmid pJ603-gag at day 0. Mice were euthanized 10 days after the last immunization and splenocytes were prepared for CMI assay. (**A**) CD4 helper cells; (**B**) CD8 T cells.

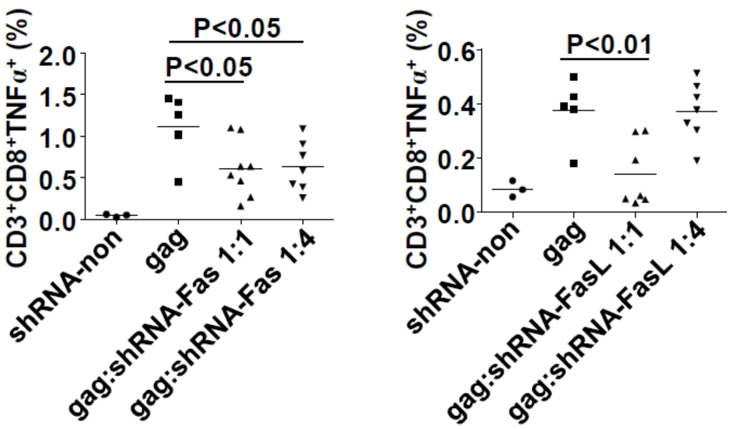

Figure S5. *In vivo* gene silencing of Fas and FasL did not improve CMI responses for SIVmac Gag. C57BL/6 mice were immunized with 2 µg or 8 µg plasmids shRNA Fas or shRNA-FasL at day 0 and day 28. Mice were euthanized 10 d after the last immunization and splenocytes were prepared for CMI assay.

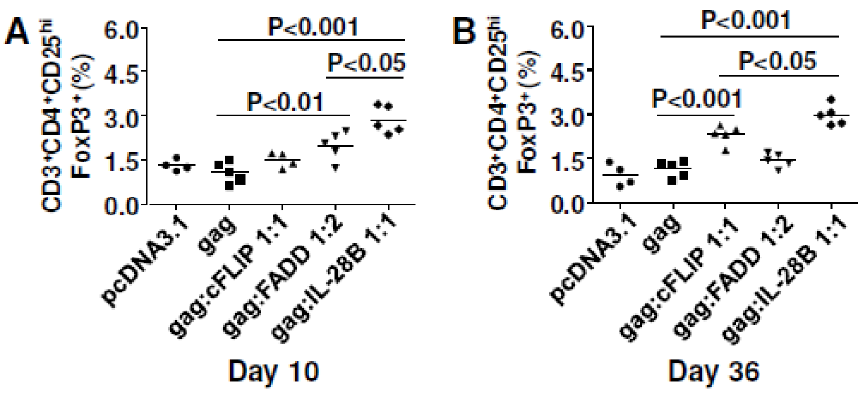

Figure S6. cFLIP, FADD and IL-28B co-delivery upregulated regulatory T (Treg) cells after DNA vaccination with SIVmac Gag. C57BL/6 mice were immunized with 2 µg plasmid pJ603-gag combined with 2 µg plasmid pJ603-cFLIP or plasmid IL-28B or 4 µg plasmid pJ603-FADD at day 0 and day 28. Mice were euthanized 10 (**A**) or 36 (**B**) days after the last immunization and splenocytes were prepared for cellular immune response assay; Data in (**A**) and (**B**) are representative of two independent experiments with similar results (n = 5 except for control group where n = 4).

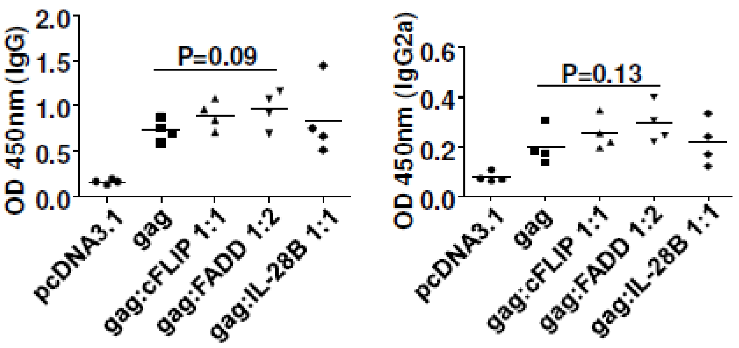

Figure S7. cFLIP, FADD and IL-28B co-delivery had little influence on humoral immune responses after DNA vaccination with SIVmac Gag. C57BL/6 mice were immunized with 2 μg plasmid pJ603-gag combined with 2 μg plasmid pJ603-cFLIP or plasmid IL-28B or 4 μg plasmid pJ603-FADD at day 0 and day 28. Sera were collected 36 days after the last immunization and diluted 100-fold before ELISA. (Left) Total IgG assay. (Right) IgG2a assay. Data are representative of two independent experiments with similar results (n = 5 except for control group where n = 4).

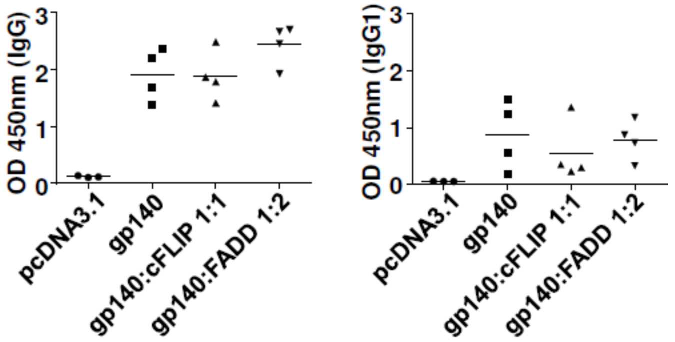

Figure S8. cFLIP and FADD co-delivery had little influence on total IgG (Left) or IgG1 (Right) titers after DNA vaccination with HIVBal gp140. C57BL/6 mice were immunized with 2 μg plasmid pJ603-gag combined with 2 μg plasmid pJ603-cFLIP or 4 μg plasmid pJ603-FADD at day 0 and day 28. Sera were collected 10 days after the last immunization and diluted 300-fold before ELISA. Data are representative of two independent experiments with similar results (n = 5 except for control group where n = 4).

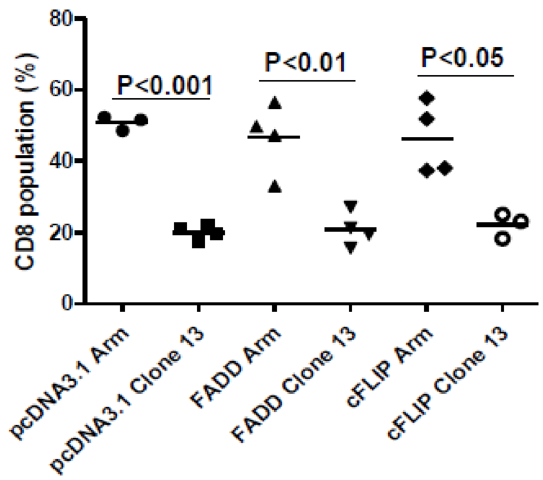

Figure S9. CD8 T cells are significantly deleted during chronic lymphocytic choriomeningitis viruse (LCMV) infection. Mice were electroporated with 2 μg plasmids of pcDNA.3.1, FADD or cFLIP on day zero (d0). On day 7, mice were infected with either 2 × 10^5 plaque-forming units (PFU) LCMV-Armstrong i.p. or 2 × 10^6 PFU LCMV-Clone13 i.v.. Nine days later, mice were sacrificed and assessed for CMI.

Table S1. Multiple Comparison Analyses of Figures 1-8. [1]

				i/j				
**Figure**	**Treatment**	**(1)**	**(2)**	**(3)**	**(4)**	**(5)**	**(6)**	**LSmean**
1B lft								
1way ANOVA	(1) Fas-/- gag		**0.0084**	**0.0003**				6.55333333
	(2) WTgag	**0.0084**		0.0629				2.95750000
	(3) pcDNA	**0.0003**	0.0629					0.08100000
1B rt								
1way ANOVA	(1) Fas-/- gag		**0.0006**	**<0.0001**				4.15333333
	(2) WTgag	**0.0006**		0.0602				1.56200000
	(3) pcDNA	**<0.0001**	0.0602					0.03525000
1C m								
1way ANOVA	(1) gag		**0.0008**	**<0.0001**	**0.0004**	**<0.0001**		0.62660000
	(2) gag Fas 1:1	**0.0008**		0.6059	0.9971	0.2150		0.23960000
	(3) gag Fas 1:2	**<0.0001**	0.6059		0.7955	0.8648		0.12780000

Table S1. *Cont.*

		i/j						
**Figure**	**Treatment**	**(1)**	**(2)**	**(3)**	**(4)**	**(5)**	**(6)**	**LSmean**
	(4) gag Fas 1:3	**0.0004**	0.9971	0.7955		0.3338		0.21400000
	(5) pcDNA	**<0.0001**	0.2150	0.8648	0.3338			0.04133333
1C lft								
1way ANOVA	(1) gag		0.9998	0.1116	0.3897	**0.0146**		3.31800000
	(2) gag Fas 1:1	0.9998		0.1481	0.4762	**0.0194**		3.1960000
	(3) gag Fas 1:2	0.1116	0.1481		0.9340	0.6536		1.30520000
	(4) gag Fas 1:3	0.3897	0.4762	0.9340		0.2865		1.90600000
	(5) pcDNA	**0.0146**	**0.0194**	0.6536	0.2865			0.08266667
1C lft								
2way ANOVA	(1) gag Fas 1:1		**0.0030**	0.1014	0.9663	**<0.0001**		3.06400000
	(2) gag Fas 1:2	**0.0030**		0.6513	**0.0004**	0.1855		1.26760000
	(3) gag Fas 1:3	0.1014	0.6513		**0.0220**	**0.0118**		1.89600000
	(4) gag	0.9663	**0.0004**	**0.0220**		**<0.0001**		3.36100000
	(5) pcDNA	**<0.0001**	0.1855	**0.0118**	**<0.0001**			0.07800000
2A lft								
1way ANOVA	(1) gag		0.9927	0.9999	**0.0215**			7.05600000
	(2)gag:shRFas 1:1	0.9727		0.9807	**0.0204**			6.59625000
	(3)gag:shRFas 1:4	0.9999	0.9807		**0.0126**			7.17857143
	(4) shRNA-non	**0.0215**	**0.0204**	**0.0126**				0.10166667
2A rt								
1way ANOVA	(1) gag		1.0000	0.9980	**0.0081**			5.36200000
	(2)gag:shRFasL 1:1	1.0000		0.9974	**0.0051**			5.36000000
	(3)gag:shRFasL 1:4	0.9980	0.9974		**0.0059**			5.16375000
	(4) shRNA-non	**0.0081**	**0.0051**	**0.0059**				0.07666667
2B lft								
1way ANOVA	(1) gag		**0.0432**	0.1196	**0.0035**			0.59420000
	(2)gag:shRFas 1:1	**0.0432**		0.9563	0.2478			0.31700000
	(3)gag:shRFas 1:4	0.1196	0.9563		0.1387			0.36128571
	(4) shRNA-non	**0.0035**	0.2478	0.1387				0.09633333
2B rt								
1way ANOVA	(1) gag		0.0689	0.5305	**0.0036**			0.48480000
	(2)gag:shRFasL 1:1	0.0689		0.5345	0.1799			0.27950000
	(3)gag:shRFasL 1:4	0.5305	0.5345		**0.0269**			0.37557143
	(4) shRNA-non	**0.0036**	0.1799	**0.0269**				0.08366667
2C lft								
1way ANOVA	(1) gag		**0.0225**	**0.0060**				1.35600000
	(2)gag:shRFas 1:1	**0.0225**		0.8613				2.24875000
	(3)gag:shRFas 1:4	**0.0060**	0.8613					2.44714286
	(4) shRNA-non	0.3473	**0.0012**	**0.0004**				0.74533333
2C rt								
1way ANOVA	(1) gag		**<0.0001**	0.9998	0.1772			1.77800000
	(2)gag:shRFasL 1:1	**<0.0001**		**<0.0001**	**<0.0001**			3.21571429

Table S1. *Cont.*

		i/j						
**Figure**	**Treatment**	**(1)**	**(2)**	**(3)**	**(4)**	**(5)**	**(6)**	**LSmean**
	(3)gag:shRFasL 1:4	0.9998	**<0.0001**		0.1484			1.76125000
	(4) shRNA-non	0.1772	**<0.0001**	0.1484				1.9500000
3A								
1way ANOVA	(1) gag: 150 μg α-FasL		**0.0051**	**0.0276**	0.3816			1.41240000
	(2)gag:150 μg isotype	**0.0051**		0.8223	**0.0003**			4.41400000
	(3) gag: 300 μg α-FasL	**0.0276**	0.8223		**0.0016**			3.77400000
	(4) pcDNA	0.3816	**0.0003**	**0.0016**				0.11725000
3B								
1way ANOVA	(1) gag: 150 μg α-FasL		**0.0036**	**0.0007**	0.3144			6.0540000
	(2)gag:150 μg isotype	**0.0036**		0.8499	**0.0002**			10.4320000
	(3) gag: 300 μg α-FasL	**0.0007**	0.8499		**<0.0001**			11.2660000
	(4) pcDNA	0.3144	**0.0002**	**<0.0001**				4.0900000
3C								
1way ANOVA	(1) gag: 150 μg α-FasL		0.1515	0.3305	0.6910			0.28440000
	(2)gag:150 μg isotype	0.1515		0.9567	**0.0253**			0.72800000
	(3) gag: 300 μg α-FasL	0.3305	0.9567		0.0623			0.62900000
	(4) pcDNA	0.6910	**0.0253**	0.0623				0.05425000
3D								
1way ANOVA	(1) gag: 150 μg α-FasL		**0.0059**	0.9181	**0.0002**			2.22600000
	(2)gag:150 μg isotype	**0.0059**		**0.0206**	0.2021			1.35200000
	(3) gag: 300 μg α-FasL	0.9181	**0.0206**		**0.0005**			2.08600000
	(4) pcDNA	**0.0002**	0.2021	**0.0005**				0.86650000
4A lft								
1way ANOVA	(1) gag		0.1242	**0.0340**	0.4133	**0.0309**		3.65800000
	(2) gag FADD1:2	0.1242		0.9624	0.9400	**0.0002**		6.42800000
	(3) gag IL28 1:1	**0.0340**	0.9624		0.6249	**<0.0001**		7.15000000
	(4) gag cFLIP 1:1	0.4133	0.9400	0.6249		**0.0011**		5.60200000
	(5) pcDNA	**0.0309**	**0.0002**	**<0.0001**	**0.0011**			0.04500000
4A lft								
2way ANOVA	(1) gag		**0.0003**	**<0.0001**	**0.0054**	**0.0004**		3.41700000
	(2) gag FADD1:2	**0.0003**		0.9785	0.8846	**<0.0001**		6.63900000
	(3) gag IL28 1:1	**<0.0001**	0.9785		0.6249	**<0.0001**		7.03700000
	(4) gag cFLIP 1:1	**0.0054**	0.8846	0.6249		**<0.0001**		5.99300000
	(5) pcDNA	**0.0004**	**<0.0001**	**<0.0001**	**0.0011**			0.04512500
4A rt								
1way ANOVA	(1) gag		0.2304	0.1695	0.8704	**0.0128**		9.8960000
	(2) gag FADD1:2	0.2304		0.9997	**0.0386**	**0.0001**		13.4600000
	(3) gag IL28 1:1	0.1695	0.9997		**0.0264**	**0.0001**		13.7600000
	(4) gag cFLIP 1:1	0.8704	**0.0386**	**0.0264**		**0.0825**		8.3280000
	(5) pcDNA	**0.0128**	**0.0001**	**0.0001**	**0.0825**			3.5475000
4A rt								
2way ANOVA	(1) gag		**0.0186**	**0.0057**	0.8698	**0.0001**		9.8880000
	(2) gag FADD1:2	**0.0186**		0.9926	**0.0012**	**0.0001**		13.4600000

Table S1. *Cont.*

		i/j						
**Figure**	**Treatment**	**(1)**	**(2)**	**(3)**	**(4)**	**(5)**	**(6)**	**LSmean**
	(3) gag IL28 1:1	**0.0057**	0.9926		**0.0003**	**0.0001**		13.9330000
	(4) gag cFLIP 1:1	0.8698	**0.0012**	**0.0003**		**0.0005**		8.8270000
	(5) pcDNA	**0.0001**	**0.0001**	**0.0001**	**0.0005**			3.5450000
4B rt								
1way ANOVA	(1) gag		**0.0672**	0.1924	0.8050	0.5893		5.94600000
	(2) gag FADD1:2	**0.0672**		0.9775	0.4238	**0.0042**		8.53200000
	(3) gag IL28 1:1	0.1924	0.9775		0.7583	**0.0135**		8.01400000
	(4) gag cFLIP 1:1	0.8050	0.4238	0.7583		0.1296		6.94000000
	(5) pcDNA	0.5893	**0.0042**	**0.0135**	0.1296			4.53000000
4B rt								
2way ANOVA	(1) gag		**0.0011**	**0.0148**	0.5148	0.1315		6.02900000
	(2) gag FADD1:2	**0.0011**		0.8960	0.0805	**0.0042**		8.53000000
	(3) gag IL28 1:1	**0.0148**	0.8960		0.4184	**0.0135**		7.99900000
	(4) gag cFLIP 1:1	0.5142	0.0805	0.4184		**0.0029**		6.96700000
	(5) pcDNA	0.1315	**0.0001**	**0.0001**	**0.0029**			4.51500000
4B lft								
1way ANOVA	(1) gag		0.1127	0.2111	0.2796	0.1640		0.82460000
	(2) gag FADD1:2	0.1127		0.9962	0.9826	**0.0010**		1.60380000
	(3) gag IL28 1:1	0.2111	0.9962		0.9998	**0.0021**		1.49620000
	(4) gag cFLIP 1:1	0.2796	0.9826	0.9998		**0.0030**		1.44300000
	(5) pcDNA	0.1640	**0.0010**	**0.0021**	**0.0030**			0.06475000
4B lft								
2way ANOVA	(1) gag		**0.0008**	**0.0040**	**0.0051**	**0.0076**		0.80430000
	(2) gag FADD1:2	**0.0008**		0.9824	0.9708	**0.0001**		1.65190000
	(3) gag IL28 1:1	**0.0040**	0.9824		1.0000	**0.0001**		1.54610000
	(4) gag cFLIP 1:1	**0.0051**	0.9708	1.0000		**0.0001**		1.53050000
	(5) pcDNA	**0.0076**	**0.0001**	**0.0001**	**0.0001**			0.06362500
5A lft								
1way ANOVA	(1) gp140		**0.0089**	**0.0180**	**0.0133**			0.71350000
	(2) gp140 :FADD 1:2	**0.0089**		0.9725	**<0.0001**			1.34750000
	(3) gp140 :cFLIP 1:1	**0.0180**	0.9725		**<0.0001**			1.28050000
	(4) pcDNA	**0.0133**	**<0.0001**	**<0.0001**				0.07033333
5A rt								
1way ANOVA	(1) gp140		**0.0146**	0.6272	**0.0150**			0.15450000
	(2) gp140 :FADD 1:2	**0.0146**		0.1100	**<0.0001**			0.23125000
	(3) gp140 :cFLIP 1:1	0.6272	0.1100		**0.0024**			0.17950000
	(4) pcDNA	**0.0150**	**<0.0001**	**0.0024**				0.07200000
5A rt								
2way ANOVA	(1) gp140		**0.0001**	**0.0272**	**0.0001**			0.15862500
	(2) gp140 :FADD 1:2	**0.0001**		0.0566	**0.0001**			0.23800000
	(3) gp140 :cFLIP 1:1	**0.0272**	0.0566		**0.0001**			0.20062500
	(4) pcDNA	**0.0001**	**0.0001**	**0.0001**				0.07050000

Table S1. *Cont.*

		i/j						
**Figure**	**Treatment**	**(1)**	**(2)**	**(3)**	**(4)**	**(5)**	**(6)**	**LSmean**
5B								
1way ANOVA	(1) gp140		0.0629	0.0820	**0.0191**			3.28000000
	(2) gp140 :FADD 1:2	0.0629		0.9984	0.7912			2.21000000
	(3) gp140 :cFLIP 1:1	0.0820	0.9984		0.7103			2.27000000
	(4) pcDNA	**0.0191**	0.7912	0.7103				1.83666667
5B								
2way ANOVA	(1) gp140		**0.0006**	**0.0007**	**0.0001**			3.36125000
	(2) gp140 :FADD 1:2	**0.0006**		0.9998	0.1081			2.22000000
	(3) gp140 :cFLIP 1:1	**0.0007**	0.9998		0.0933			2.24000000
	(4) pcDNA	**0.0001**	0.1081	0.0933				1.58000000
5C								
1way ANOVA	(1) gp140		**0.0264**	0.8048	0.8825			0.18100000
	(2) gp140 :FADD 1:2	**0.0264**		0.1167	**0.0119**			0.53300000
	(3) gp140 :cFLIP 1:1	0.8048	0.1167		0.4352			0.27450000
	(4) pcDNA	0.8825	**0.0119**	0.4352				0.09900000
5C								
2way ANOVA	(1) gp140		**0.0001**	0.1699	0.5144			0.18275000
	(2) gp140 :FADD 1:2	**0.0001**		**0.0020**	**0.0001**			0.54462500
	(3) gp140 :cFLIP 1:1	0.1699	**0.0020**		**0.0122**			0.30662500
	(4) pcDNA	0.5144	**0.0001**	**0.0122**				0.09533333
6A								
1way ANOVA	(1) gag		0.4292	0.9972				5.31000000
	(2) gag shRCasp8	0.4292		0.2981				8.15250000
	(3) gag shRFADD	0.9972	0.2981					4.97600000
	(4) shRNA-non	0.0821	**0.0066**	0.0902				0.07800000
6B								
1way ANOVA	(1) gag		0.2994	0.1068	0.5994			0.65625000
	(2) gag shRCasp8	0.2994		0.9385	**0.0492**			1.44000000
	(3) gag shRFADD	0.1068	0.9385		**0.0155**			1.67020000
	(4) shRNA-non	0.5994	**0.0492**	**0.0155**				0.07600000
6C								
1way ANOVA	1) gag		0.1718	0.4346	0.9006			9.7475000
	2) gag shRCasp8	0.1718		0.8608	0.5379			14.6100000
	3) gag shRFADD	0.4346	0.8608		0.8884			12.9860000
	4) shRNA-non	0.9006	0.5379	0.8884				11.3666667
7A lft								
1way ANOVA	(1) FADD Arm		0.2520	0.9986	0.9917	0.9449	0.9965	1.94000000
	(2) FADD Clone13	0.2520		0.4359	0.6369	0.0746	0.4809	1.01950000
	(3) cFLIP Arm	0.9986	0.4359		0.9999	0.8075	1.0000	1.78250000
	(4) cFLIP Clone13	0.9917	0.6369	0.9999		0.7490	1.0000	1.69333333
	(5) pcDNA Arm	0.9449	0.0746	0.8075	0.7490		0.7689	2.32333333
	(6) pcDNA Clone13	0.9965	0.4809	1.0000	1.0000	0.7689		1.75000000

Table S1. *Cont.*

Figure	Treatment	(1)	(2)	(3)	(4)	(5)	(6)	LSmean
		i/j						
7A rt	(1) FADD Arm		0.1792	0.4697	0.9984	0.9900	0.6762	0.79400000
1way ANOVA	(2) FADD Clone13	0.1792		0.9831	0.4236	0.5340	0.9043	0.33125000
	(3) cFLIP Arm	0.4697	0.9831		0.7780	0.8703	0.9991	0.45450000
	(4) cFLIP Clone13	0.9984	0.4236			1.0000	0.9180	0.71466667
	(5) pcDNA Arm	0.9900	0.5340	0.8703	1.0000		0.9664	0.67566667
	(6) pcDNA Clone13	0.6762	0.9043	0.9991	0.9180	0.9664		0.51950000
7B lft								
1way ANOVA	(1) FADD Arm		**0.0021**	0.7171	0.0638	0.9849	0.1136	91.8250000
	(2) FADD Clone13	**0.0021**		**0.0340**	0.7326	**0.0153**	0.3509	83.6250000
	(3) cFLIP Arm	0.7171	**0.0340**		0.5034	0.9839	0.7500	89.4250000
	(4) cFLIP Clone13	0.0638	0.7236	0.5034		0.2559	0.9944	86.1666667
	(5) pcDNA Arm	0.9849	**0.0153**	0.9839	0.2559		0.4271	90.6333333
	(6) pcDNA Clone13	0.1136	0.3509	0.7500	0.9944	0.4271		87.1250000
7B rt								
1way ANOVA	(1) FADD Arm		0.9032	0.8686	0.5552	0.9980	1.0000	3.59000000
	(2) FADD Clone13	0.9032		1.0000	0.9729	0.9941	0.9519	4.29750000
	(3) cFLIP Arm	0.8686	1.0000		0.9839	0.9886	0.9277	4.36000000
	(4) cFLIP Clone13	0.5552	0.9729	0.9839		0.8351	0.6466	4.85333333
	(5) pcDNA Arm	0.9980	0.9941	0.9886	0.8351		0.9998	3.90333333
	(6) pcDNA Clone13	1.0000	0.9519	0.9277	0.6466	0.9998		3.70500000
8								
1way ANOVA	(1) FADD Arm		1.0000	1.0000	1.0000	1.0000	**0.0001**	3.6250
	(2) FADD Clone13	1.0000		1.0000	1.0000	1.0000	**0.0001**	59.0000
	(3) cFLIP Arm	1.0000	1.0000		1.0000	1.0000	**0.0001**	4.5000
	(4) cFLIP Clone13	1.0000	1.0000	1.0000		1.0000	**0.0001**	71.4286
	(5) pcDNA Arm	1.0000	1.0000	1.0000	1.0000		**0.0001**	3.8333
	(6) pcDNA Clone13	**0.0001**	**0.0001**	**0.0001**	**0.0001**	**0.0001**		49,750.0

[1] Figures that have been subjected to the conventional GraphPad Prism analysis have also been subjected to a Tukey-Kramer adjustment for multiple comparisons. The data are tabulated to represent the type of treatment (Treatment), the Least squares mean (LSmean) for each treatment, and the pairwise comparisons of least squares means (i/j) with bolded numbers for significant values. Single conclusive experiments were analyzed by one-way ANOVA, as indicated in Table S1; however, in cases where a single experiment was inconclusive, data from repeat experiments was analyzed by 2-way ANOVA and tabulated after the 1-way ANOVA analysis (e.g. Figure 4B-left panel does not show a significant difference between the tetramer staining of gag-vaccinated mouse splenocytes and tetramer staining of splenocytes from mice co-inoculated with cFLIP or FADD, therefore, the Table also shows a 2-way ANOVA, that supports a significant difference). The conclusions in our discussion are supported by the additional multiple comparison analysis.
